# A simplified MALDI-TOF MS method for rapid fluconazole susceptibility testing in Candida species

**DOI:** 10.1099/jmm.0.002172

**Published:** 2026-06-15

**Authors:** Bárbara Cipulo Legabão, João Pedro Guedes Xavier, Ana Luisa Perini Leme Giordano, Lais Pontes, Mauro Luiz Brandão Junior, Angélica Zaninelli Schreiber

**Affiliations:** 1 Pathology Department – School of Medical Sciences - University of Campinas, School of Medical Sciences, Campinas, São Paulo, Brazil; 2 Brazilian Synchrotron Light Laboratory (LNLS), Brazilian Center for Research in Energy and Materials (CNPEM), Campinas, São Paulo, Brazil

**Keywords:** antifungal susceptibility testing, Candida, Fluconazole, MALDI-TOF MS, resistance

## Abstract

**Introduction.** Matrix-assisted laser desorption/ionization time-of-flight mass spectrometry (MALDI-TOF MS) is widely used for rapid micro-organism identification and has recently been explored for antifungal susceptibility testing (AFST).

**Hypothesis.** Although MALDI-TOF MS has emerged as a promising tool for AFST, simplified and clinically applicable strategies for rapid fluconazole (FLZ) susceptibility detection in *Candida* spp. remain insufficiently validated. We hypothesized that a streamlined AFST-MS approach would demonstrate good categorical agreement (CA) with the European Committee on Antimicrobial Susceptibility Testing (EUCAST) reference method while significantly reducing turnaround time.

**Aim.** To establish a simplified MALDI-TOF MS-based AFST approach for detecting FLZ resistance in *Candida* species.

**Methodology.** Fifty-one clinical isolates and reference strains were incubated for 3 h in the presence of FLZ at two concentrations (32 and 4 µg ml^−1^) and in drug-free controls. Spectral profiles were compared with the EUCAST reference method.

**Results.** Overall CA between AFST-MS and EUCAST was 85.2% (κ=0.7306). Species-specific accuracy was 100% for *Candida auris, Pichia kudriavzevii* (formerly *Candida krusei*), *Candida tropicalis* and *Candida parapsilosis*; 92.9% for *Candida albicans* and 40% for *Nakaseomyces glabrata* (formerly *Candida glabrata*); however, these estimates should be interpreted cautiously given the limited number of isolates per species. All discrepancies were minor errors, with no major or very major errors observed. The method reduced analysis time from 24 to 3 h and enabled presumptive FLZ susceptibility detection with good overall agreement with the reference method

**Conclusion.** These findings support the potential of MALDI-TOF MS as a rapid adjunct tool for antifungal susceptibility assessment and may contribute to earlier therapeutic decision-making.

## Introduction

Invasive fungal infections caused by *Candida* species represent a major cause of morbidity and mortality in hospitalized patients, especially those who are immunocompromised [1]. In recent years, the incidence of these infections has increased, with a higher prevalence of non-albicans species such as *Candida auris, Candida tropicalis* and *Nakaseomyces glabrata* (formerly *Candida glabrata*), often associated with antifungal resistance and poorer clinical outcomes [2].

Fluconazole (FLZ) is an azole antifungal widely used for the treatment and prophylaxis of *Candida* infections due to its favourable tolerability profile, excellent oral bioavailability and extensive tissue penetration [3]. However, prolonged or repeated exposure may select for resistant strains, reducing treatment efficacy and increasing the risk of therapeutic failure [4]. This trend is particularly concerning given the limited therapeutic options available [4]. The development of resistance is driven by factors such as the excessive use of antifungals in clinical practice [5]. Delayed diagnosis further exacerbates this problem, underscoring the need for rapid and accurate fungal identification coupled with susceptibility testing to guide timely and targeted therapy, while safeguarding the effectiveness of first-line drugs such as FLZ [5]. Conventional susceptibility tests, such as broth microdilution assays standardized by the Clinical and Laboratory Standards Institute (CLSI) and the European Committee on Antimicrobial Susceptibility Testing (EUCAST) [6,7], remain the reference standard but are labour-intensive, time-consuming and reliant on subjective visual interpretation, which hampers prompt clinical decision-making [8].

Matrix-assisted laser desorption/ionization time-of-flight mass spectrometry (MALDI-TOF MS) has emerged as a tool to streamline laboratory workflows. Originally developed for rapid and accurate microbial identification, the technique has since been adapted for antimicrobial susceptibility testing [9,11]. In previous work, we applied the MALDI-TOF MS-based method to assess azole susceptibility in *Candida* and *Aspergillus* using the composite correlation index (CCI) [12], a statistical measure of spectral similarity. This method determines the minimal profile change concentration (MPCC), defined as the lowest drug concentration that alters the microbial protein profile after 15 h of exposure. The MPCC correlates with the minimum inhibitory concentration (MIC), enabling direct comparison with CLSI and EUCAST reference standards methods [10,12, 13]. Although this strategy shortens the time to results relative to conventional methods, its applicability in routine diagnostics still offers limited advantages over more recent strategies.

In this work, we aimed to enhance clinical utility by developing a modified MALDI-TOF MS-based protocol (AFST-MS) that reduces FLZ exposure to just 3 h. By employing fixed drug concentrations, this approach allows rapid and reliable discrimination between FLZ-susceptible and -resistant isolates.

## Methods

### Reactivation and selection of isolates

This study included 51 clinical isolates of *Candida* spp., comprising 14 *Candida albicans*, 10 *N. glabrata*, 9 *C*. *auris*, 8 *C*. *tropicalis*, 6 *Pichia kudriavzevii* and 4 *Candida parapsilosis*. Reference strains (*C. albican*s ATCC 90028, *C. parapsilosis* ATCC 22019 and *P. kudriavzevii* ATCC 6258) were included for quality control.

Clinical isolates, collected between 2017 and 2025, were obtained from the culture collection of the Laboratory of Fungal Investigation at the University of Campinas (LIF-Unicamp). Isolates were subcultured on Sabouraud dextrose agar (SDA) and incubated at 35 °C. After 24 h of incubation, species identification was confirmed by MALDI-TOF MS using a Microflex LT^®^ system (Bruker Daltonics, Germany) operated with FlexControl 3.4 software.

### AFST by the EUCAST reference method

MICs were determined by broth microdilution following the EUCAST standard (E.Def 7.4) [6]. After growth on SDA, inocula were prepared in sterile distilled water and adjusted to a 0.5 McFarland standard using a spectrophotometer at 530 nm. FLZ was tested over a concentration range of 0.06–32 µg ml^−1^.

Quality control strains recommended by EUCAST were included in each assay. Microdilution plates were incubated at 35 °C, and MIC values were determined after 24 h using an Epoch BioTek^®^ microplate spectrophotometer at 404 nm. Isolates were categorized as susceptible, intermediate or resistant according to EUCAST clinical breakpoints.

### AFST by MALDI-TOF MS (AFST-MS)

AFST-MS analysis was adapted from the method described by Giordano *et al*. [12], with modifications to reduce incubation time. Briefly, isolates were exposed to FLZ at three concentrations: 32 µg ml^−1^ (high), 4 µg ml^−1^ (intermediate) and 0 µg ml^−1^ (drug-free control). Samples were incubated at 35 °C for 3 h under constant agitation using an l-Mixer 360 (Loccus, Brazil) set to mode F6 (speed 54, frequency 1). The maximum and intermediate concentrations were selected based on the EUCAST cutoff points for FLZ. All experiments were performed in triplicate.

Following incubation, cells were washed sequentially with distilled water and 75% ethanol to remove potential interferents. Protein extraction was carried out using 70% formic acid followed by acetonitrile and centrifugation. Aliquots (1 µl) of the resulting supernatant were spotted onto a polished stainless steel target plate and air-dried at room temperature.

The matrix solution consisted of α-cyano-4-hydroxycinnamic acid (HCCA) prepared in an organic solvent composed of 475 µl of sterile distilled water, 25 µl of 100% trifluoroacetic acid and 500 µl of acetonitrile. A final matrix solution was obtained by mixing 250 µl of this solvent with 80 µl of acetonitrile. 1 µl of matrix was applied to each spot, followed by air-drying prior to analysis.

Spectra were acquired in the mass range of 2–20 kDa using FlexControl software. External calibration was performed using the Bruker Bacterial Test Standard (BTS), according to the manufacturer’s instructions. The BTS, containing proteins with known mass peaks (3,600–20,000 Da), was reconstituted, applied to the target plate, overlaid with HCCA matrix, and analysed prior to sample acquisition to ensure instrument performance and mass accuracy.

### MALDI-TOF MS data analysis

Spectral acquisition was performed using FlexControl 3.4 software (Bruker Daltonics, Germany). The resulting spectra were subsequently processed using FlexAnalysis software (Bruker Daltonics, Germany), where preprocessing steps included spectral smoothing and baseline subtraction to improve signal quality and facilitate interpretation. Default processing parameters provided by the software were applied.

Processed spectra were then analysed using Biotyper 3.0 software (Bruker Daltonics, Bremen, Germany). Spectral similarity was assessed using the CCI, with pairwise comparisons performed between spectra obtained under different antifungal conditions, including the two extreme concentrations (0 and 32 µg ml^−1^). The resulting similarity matrices were visualized as heatmaps, in which warm colours indicate high similarity and cool colours indicate low similarity.

**Fig. 1.**
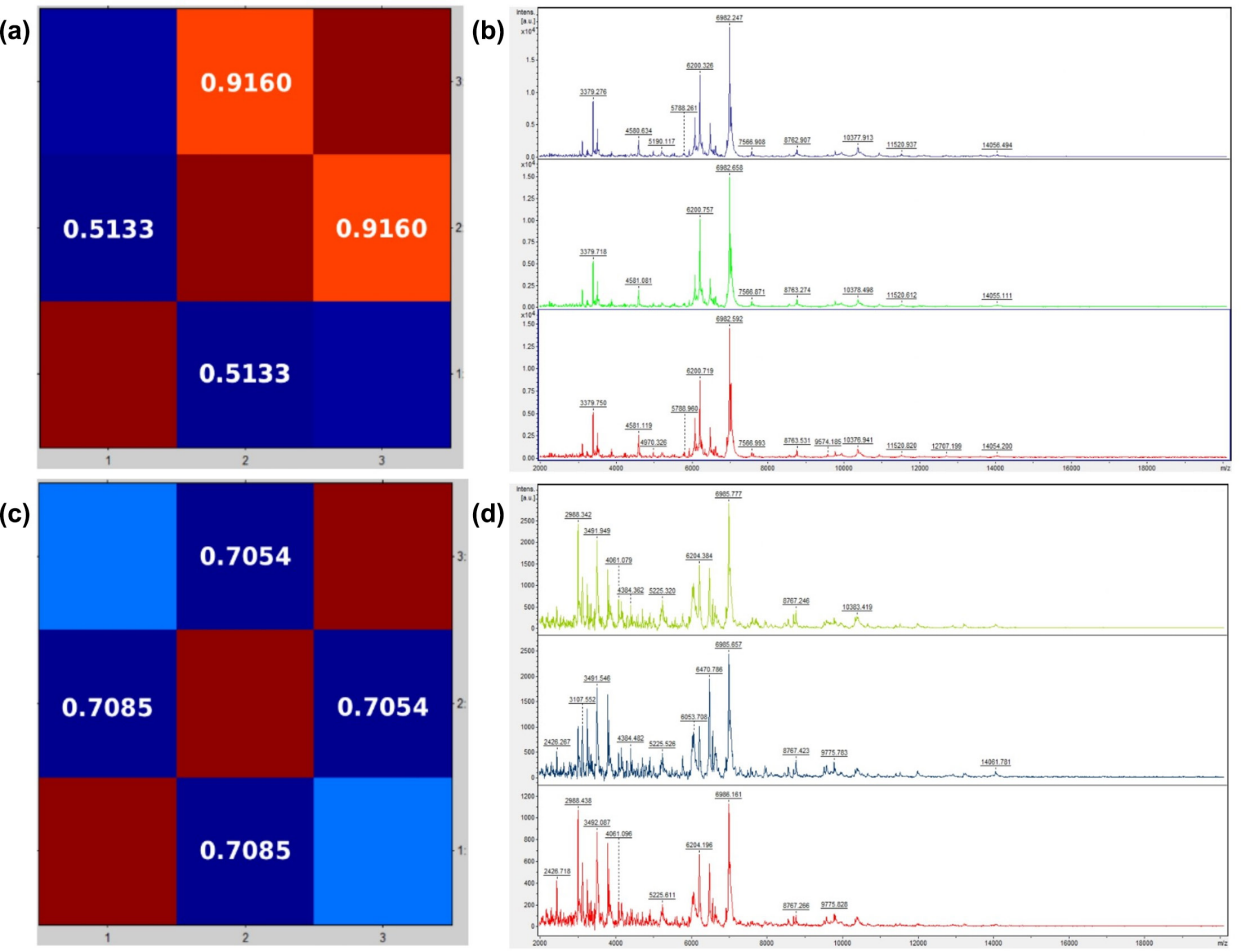
Representative examples of susceptibility categorization by AFST-MS in *C. albicans* isolates. Subfigures (a) and (c) show correlation matrices derived from the CCI of MALDI-TOF MS spectra. Susceptibility classification is based on the comparison between maximum CCI values (4 vs 32 μg ml^−1^) and null CCI values (4 vs 0 μg ml^−1^), which are indicated in the heatmaps. Isolate LIF 15272 (a) was classified as susceptible to FLZ, as the CCI between 4 and 32 μg ml^−1^ was higher than that between 4 and 0 μg ml^−1^ (CCI = 0.9160>0.5133). In contrast, isolate LIF E10 (c), previously identified as carrying mutations in the ERG11 gene, was classified as resistant, as the CCI between 4 and 32 μg ml^−1^ was lower than that between 4 and 0 μg ml^−1^ (CCI=0.7054<0.7085). Subfigures (b) and (d) present the corresponding spectra obtained for each tested concentration (0, 4 and 32 μg ml^−1^), visualized using FlexAnalysis software (Bruker Daltonics, Germany). Minimal visible alterations in protein profiles highlight the importance of CCI-based analysis for discrimination.

Each isolate was classified as susceptible or resistant based on mean CCI values. Isolates were considered susceptible when similarity between spectra at 4 and 32 µg ml^−1^ exceeded that between 4 and 0 µg ml^−1^, and resistant when the opposite pattern was observed (Fig. 1).

Agreement between AFST-MS and the EUCAST reference method was evaluated using essential agreement, defined as MIC values within ±2 dilutions and assessed by the Wilcoxon signed-rank test, and categorical agreement (CA), calculated using Cohen’s weighted κ coefficient with 95% confidence intervals.

Error rates were classified according to standard definitions: very major errors (VME), when EUCAST-resistant isolates were classified as susceptible by AFST-MS; major errors (MEs), when susceptible isolates were classified as resistant and minor errors (mEs), when intermediate isolates were classified as either susceptible or resistant.

To assess internal reproducibility, mean maximum CCI values (4 vs 32 µg ml^−1^) were compared with mean null CCI values (4 vs 0 µg ml^−1^) obtained from triplicate measurements. Results were expressed as mean±sd, and comparisons were performed using an unpaired t-test as an exploratory assessment of consistency across replicates (*P*<0.05). Detailed results are provided in Table S1, available in the online Supplementary Material.

All statistical analyses were performed using SAS System for Windows, version 9.4 (SAS Institute Inc., Cary, NC, USA, 2023).

## Results

The range of FLZ concentrations was selected to identify an intermediate concentration applicable across different *Candida* species, allowing comparison with spectra obtained at the extreme conditions (0 and 32 µg ml^−1^). The concentration of 4 µg ml^−1^ was chosen as the intermediate point, as it corresponds to the EUCAST resistance breakpoint for most of the species evaluated.

AFST-MS correlated protein spectra from each FLZ condition using the CCI tool, generating similarity matrices visualized as heatmaps. In these representations, warm colours indicate high similarity and cool colours indicate low similarity between spectra. Fig. 1 shows a representative example of susceptibility classification based on heatmap analysis. Susceptible isolates exhibited higher spectral similarity between intermediate and high drug concentrations, whereas resistant isolates showed greater similarity between intermediate and drug-free conditions, supporting the discriminatory capacity of the method.

Table 1 summarizes MIC values, EUCAST-based categories (S, I or R), and the corresponding AFST-MS results (S/R), along with CA between the methods. Overall, a high level of agreement was observed, although discrepancies were identified in specific species, particularly *N. glabrata*, contributing to reduced CA. Most discrepancies were classified as mE, mainly associated with differences related to the intermediate category.

**Table 1.** FLZ susceptibility of *Candida* isolates by broth microdilution and AFST-MS, with CA

Species	Strain designation	EUCAST		AFST-MS	CA%
		MIC (µg ml^−1^)	S, I or R category		
*C. albicans* [14]	LIF 14297	0.5	S	S	92.9
	LIF 14306	1	S	S	
	LIF 14526	0.25	S	S	
	LIF 15234	0.5	S	S	
	LIF 12560	4	I	R	
	LIF 14112	0.25	S	S	
	LIF 14200	0.25	S	S	
	LIF 14211	0.125	S	S	
	LIF 14004	0.25	S	S	
	LIF 15188	0.25	S	S	
	LIF 15272	0.125	S	S	
	LIF 15355	0.25	S	S	
	LIF 16543	32	R	R	
	LIF E10	32	R	R	
*C. parapsilosis* [4]	LIF 14451	0.5	S	S	100
	LIF 14447	1	S	S	
	LIF 15134	1	S	S	
	LIF 16747	0.5	S	S	
*C. tropicalis* [8]	LIF 14464	2	S	S	100
	LIF 14529	0.25	S	S	
	LIF 14846	0.5	S	S	
	LIF 15292	0.125	S	S	
	LIF 16453	8	R	R	
	LIF 16496	32	R	R	
	LIF 16924	32	R	R	
	LIF 16903	32	R	R	
*N. glabrata* [10]	LIF 14103	8	I	R	40
	LIF 14119	8	I	R	
	LIF 14330	32	R	R	
	LIF 14500	1	I	S	
	LIF 14821	8	I	R	
	LIF 14840	32	R	R	
	LIF 15888	16	I	R	
	LIF 16987	32	R	R	
	LIF 16995	32	R	R	
	LIF 16574	4	I	R	
*C. auris* [9]	LIF 16597	1	S	S	100
	LIF 16605–1	1	S	S	
	LIF 16605–2	2	S	S	
	LIF 16606–1	2	S	S	
	LIF 16606–2	1	S	S	
	LIF 16607	2	S	S	
	LIF 16610	0.25	S	S	
	LIF 16615	0.125	S	S	
	LIF 16619	0.25	S	S	
*P. kudriavzevii* [6]	LIF 16152	32	R	R	100
	LIF 16234	32	R	R	
	LIF 16602	32	R	R	
	LIF 15557	32	R	R	
	LIF 15506	16	R	R	
	LIF 16395	16	R	R	
*C. albicans* ATCC 90028		0.25	S	S	100
*C. parapsilosis* ATCC 22019		1	S	S	100
*P. kudriavzevii* ATCC 6258		32	R	R	100

AFST-MS, MALDI-TOF MS-based antifungal susceptibility testing; CA, categorical agreement; EUCAST, European Committee on Antimicrobial Susceptibility Testing; FLZ, fluconazole; I, intermediary; MIC, minimum inhibitory concentration; R, resistant; S, susceptible.

Agreement between EUCAST and AFST-MS varied among species. CA reached 100% for *C. auris*, *P. kudriavzevii*, *C. parapsilosis* and *C. tropicalis*; however, these estimates are limited by the small number of isolates per species. *C. albicans* showed a CA of 92.9%, whereas *N. glabrata* showed lower agreement (40%). The overall weighted κ coefficient was 0.7306 (95% CI: 0.0728–0.8732), corresponding to an overall CA of 85.2%.

Most discrepancies were associated with isolates categorized as intermediate by EUCAST, which were predominantly classified as resistant by AFST-MS. One exception was an *N. glabrata* isolate (LIF 14500), classified as intermediate by EUCAST and susceptible by AFST-MS. This pattern was observed in six *N. glabrata* isolates and one *C. albicans* isolate, contributing to reduced agreement in these species. No MEs or VMEs were identified.

Mean maximum CCI values (4 vs 32 µg ml^−1^) were compared with mean null CCI values (4 vs 0 µg ml^−1^) obtained from triplicate measurements Table S1. Several isolates showed statistically significant differences (*P*<0.05), supporting the reproducibility and discriminatory capacity of the method.

**Fig. 2.**
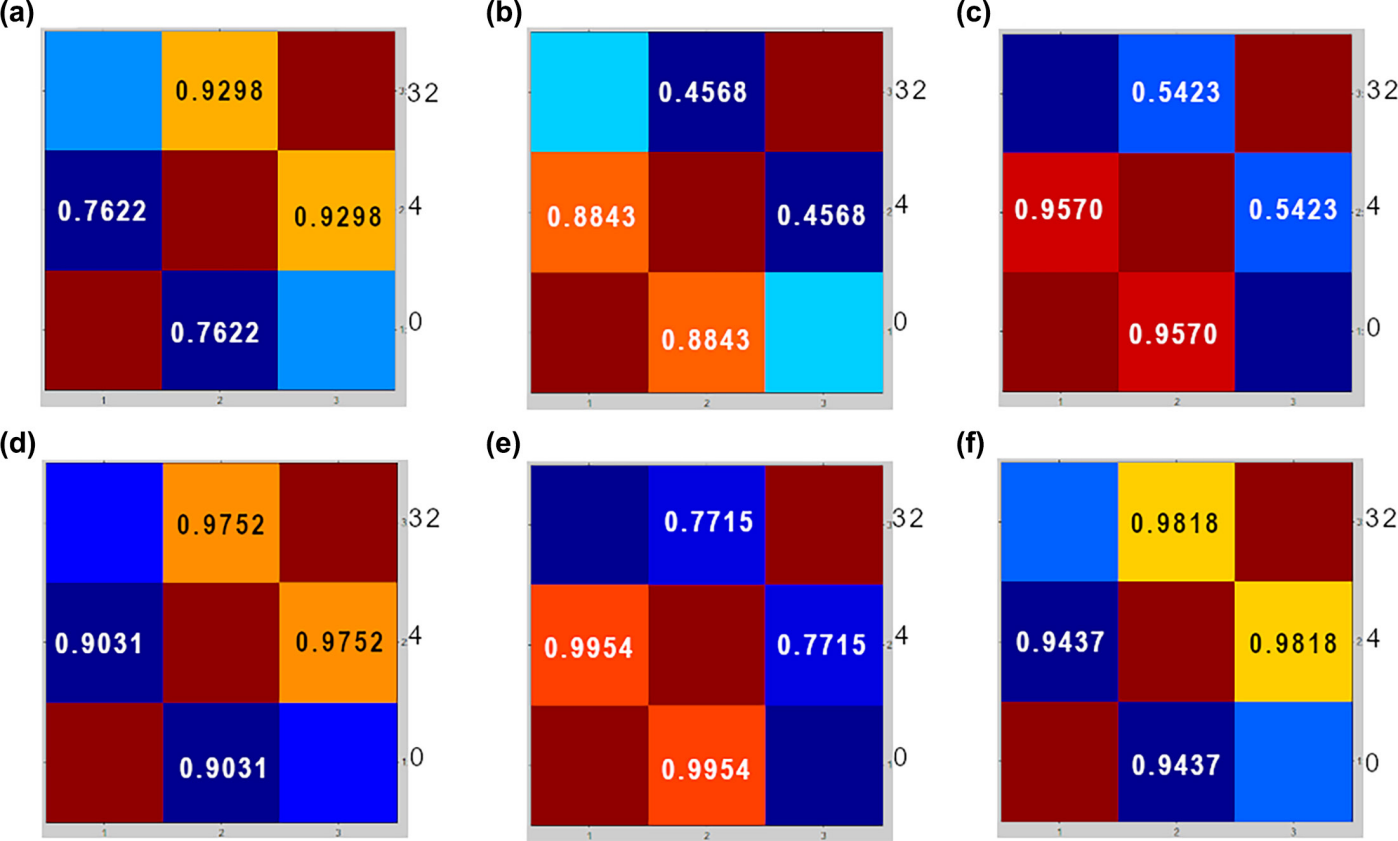
Heatmaps obtained using simplified AFST-MS for different *Candida* species exposed to FLZ. Each panel shows a correlation matrix of CCI values derived from protein spectra obtained at 0, 4 and 32 µg ml^−1^. Susceptibility classification is based on the relative comparison between maximum (4 vs 32 µg ml^−1^) and null (4 vs 0 µg ml^−1^) CCI values. The examples represent different susceptibility profiles: (**a**) LIF 14451 (*C. parapsilosis*), susceptible; (**b**) LIF 14330 (*N. glabrata*), resistant; (**c**) LIF 16496 (*C. tropicalis*), resistant; (**d**) LIF 16606–2 (*C. auris*), susceptible; (**e**) LIF 16234 (*P. kudriavzevii*), intrinsically resistant to FLZ and (**f**) *C. albicans* ATCC 90028, susceptible. Consistent spectral similarity patterns across panels support the applicability of the method for distinguishing susceptibility profiles.

Fig. 2 presents representative heatmaps for each *Candida* species, illustrating consistent spectral similarity patterns across species and supporting the robustness of the method.

**Fig. 3.**
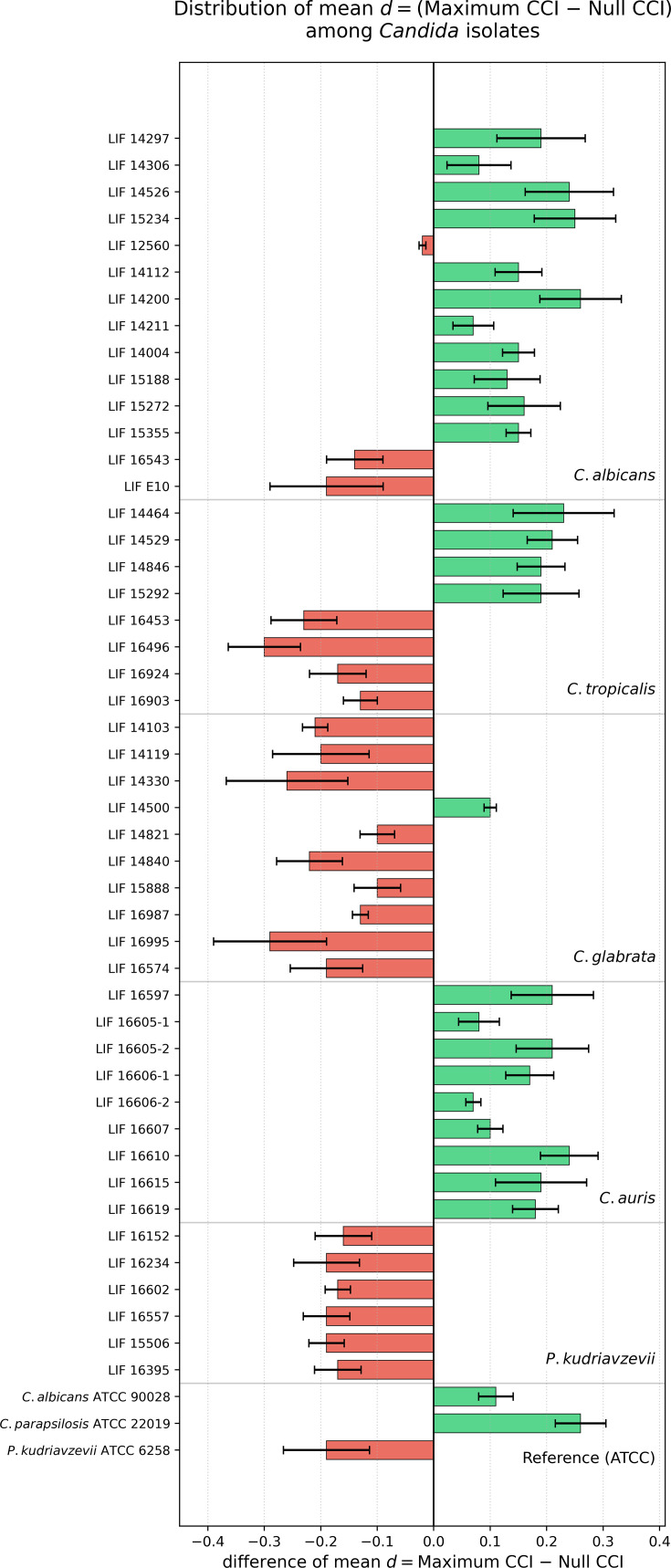
Distribution of the derived variable (d) across *Candida* isolates. The variable d was defined as the difference between the mean CCI values obtained for the comparisons (4 vs 32 µg ml^−1^) and (4 vs 0 µg ml^−1^) for each isolate. Positive values indicate greater similarity to the high-concentration condition, whereas negative values indicate greater similarity to the untreated condition. Bars represent mean d values for each isolate, with error bars indicating variability across measurements. Green and red bars correspond to susceptible and resistant isolates, respectively.

Fig. 3 summarizes the difference between mean maximum and null CCI values across isolates. Susceptible isolates predominantly showed positive d values, whereas resistant isolates showed negative values, consistent with the classification pattern described above. This distribution supports the reproducibility of the method across different *Candida* species.

## Discussion

Our results demonstrate that MALDI-TOF MS can provide a rapid and reliable assessment of FLZ susceptibility in *Candida* species. In addition to species-level identification, this approach enables early detection of susceptibility profiles, which may support timely therapeutic decisions. A key advantage is the reduction in analysis time from 24 to 3 h, which may improve clinical management, particularly in critically ill patients.

Although EUCAST and CLSI reference methods remain the standard for antifungal susceptibility testing [6,7], their time-consuming nature limits rapid clinical decision-making [8,14]. In this context, MALDI-TOF MS represents a practical alternative for accelerating susceptibility assessment [15,16].

This study included fungal isolates with mutations associated with drug resistance, previously identified in studies by our group [17,18]. The *C. albicans* strains LIF-E10 and LIF-12560 harbour mutations in the *ERG11* gene conferring resistance to FLZ: G448V and G464S in LIF-E10 and E116D, T128K, E266D and A298V in LIF-12560. The results obtained by AFST-MS were consistent with the previously characterized resistance profiles, showing susceptibility categories compatible with the high MIC values. This finding demonstrates the ability of AFST-MS to reflect the resistance profile of the isolates, validating its applicability in detecting resistant phenotypes. In addition, reproducibility analysis based on triplicate measurements (Table S2) showed consistent differences between maximum and null CCI values for several isolates, supporting the method’s capacity to discriminate between susceptibility profiles. Although statistical comparisons of CCI values were exploratory, they provided additional support for the discriminatory capacity of the method. Although threshold-based approaches, such as Receiver Operating Characteristic (ROC) curve analysis, are commonly used to define optimal cutoffs in diagnostic and susceptibility studies, their application is not conceptually aligned with the present methodology. The CCI is a relative, isolate-dependent metric, in which each strain serves as its own internal control. Therefore, the use of a global cutoff would disregard isolate-specific variability and move away from the reference-based framework that underlies standardized susceptibility testing. Future studies including larger and more heterogeneous datasets may allow the evaluation of threshold-based approaches and their integration with this methodology. Consistent with this observation, this pattern is further supported by the distribution of the derived variable (d) (Fig. 3), which shows a consistent separation between susceptible and resistant isolates across species.

Vella *et al.* [19] conducted a study using the AFST-MS method to assess the susceptibility of *C. albicans* to caspofungin, including isolates with fks1 gene mutations. The method correctly classified susceptibility in over 90% of isolates, suggesting its potential applicability to other antifungal classes, such as echinocandins. Supporting these findings, Saracli *et al.* [13] tested isolates of *C. albicans*, *C. tropicalis* and *P. kudriavzevii* against triazoles using the same simplified AFST-MS protocol and obtained results consistent with ours. More recently, Delavy *et al*. [20] exposed *C. albicans* to three FLZ concentrations, with or without cyclosporine, for 3 h using a machine-learning-based approach. They achieved an overall accuracy of 85.71%, further supporting the potential of rapid AFST-MS. In this context, future studies applying this methodology to antifungal agents from other drug classes may further expand its applicability and clinical relevance.

Compared to previously described MALDI-TOF MS-based AFST approaches, the present method reduces incubation time while maintaining acceptable agreement. In contrast to CCI-based MPCC strategies, which require longer incubation periods and multiple antifungal concentrations, our approach relies on a simplified workflow. Similarly, although machine-learning-based methods, such as those proposed by Delavy *et al*. [20], may achieve comparable accuracy, their implementation may be limited by the need for specialized computational infrastructure. A comparison of these approaches is summarized in Table S2.

Among the isolates tested, 85.2% were correctly classified. The overall agreement between AFST-MS and the EUCAST method was good (CA: 85.2%; weighted κ: 0.73, 95% CI: 0.07–0.87), supporting its potential as a rapid screening tool. However, the relatively wide confidence interval and the limited number of isolates for some species (e.g. *n*=4 for *C. parapsilosis*) suggest that species-specific performance estimates are limited by the small number of isolates for some species. Discrepancies (13%) were mainly observed in *C. albicans* and *N. glabrata*, with notably lower performance for *N. glabrata* (40% CA), representing an important limitation given its clinical relevance in azole resistance.

The reduced performance observed for *N. glabrata* may be explained by species-specific biological characteristics. This species often shows dose-dependent susceptibility to FLZ (CLSI SDD category, corresponding to the EUCAST ‘susceptible, increased exposure’ [I] category), indicating variable susceptibility levels rather than clearly defined categories [6,7]. According to EUCAST, the entire wild-type population of *N. glabrata* falls within the I category for FLZ, and isolates with MICs>16 mg l^−1^ should be interpreted as resistant. This narrow and shifted susceptibility distribution may contribute to heterogeneous stress responses and reduced discrimination of MALDI-TOF MS spectral profiles. Azole resistance in *N. glabrata* is mainly driven by activating mutations in PDR1, leading to increased expression of efflux pumps such as CDR1 and CDR2 [21,22]. Different PDR1 mutations can induce distinct transcriptional profiles, resulting in heterogeneous phenotypic responses [23,24].

This heterogeneity may affect cellular protein expression and the response to antifungal exposure, potentially impacting MALDI-TOF MS-based classification [25]. In addition, residual growth under azole exposure (trailing effect), a well-described phenomenon in *Candida* spp., may further complicate susceptibility assessment [25,26]. Together, these factors may explain the lower agreement observed for *N. glabrata*.

Although all discrepancies were classified as mE, this should be interpreted in the context of the binary AFST-MS classification. In this approach, isolates are categorized only as susceptible or resistant, without an intermediate category. This characteristic may also influence the interpretation of performance metrics, as the absence of an intermediate category can contribute to an apparent increase in CA. A similar limitation has been reported in previous studies, such as that by Saracli *et al*. [13], in which isolates classified as susceptible dose-dependent (SDD) by the CLSI method had to be reassigned due to the lack of an equivalent category in MALDI-TOF MS-based testing. In our study, most isolates categorized as intermediate by EUCAST were classified as resistant by AFST-MS, reflecting a conservative tendency of the method. From a clinical perspective, this approach may be advantageous, as isolates requiring increased drug exposure or presenting borderline susceptibility are less likely to be misclassified as fully susceptible, potentially reducing the risk of therapeutic failure.

Similar methodological constraints have been described in studies by Vella *et al*. [19] and Delavy *et al*. [20], which also employed simplified AFST-MS approaches based on the use of a limited number of predefined antifungal concentrations and binary outputs. These strategies are designed to provide rapid, actionable results rather than to fully reproduce the categorical structure of reference methods such as EUCAST or CLSI. Therefore, the absence of an intermediate category should be considered an intrinsic feature of the method and interpreted within the context of its intended clinical application as a rapid, presumptive tool for antifungal susceptibility assessment.

These findings indicate that the method may be suitable for some species, such as *C. albicans* and *C. auris*, but less reliable for others, particularly *N. glabrata*. In the case of the *C. albicans* isolate (LIF 12560), the discrepancy may be attributed to experimental variation, as the majority of isolates from this species were correctly classified.

Despite these promising results, some limitations should be acknowledged. The relatively small number of isolates for certain species may limit the generalizability of our findings. In addition, other clinically relevant fungi, such as *Aspergillus* spp., should also be explored, especially since conventional susceptibility methods can take up to 48 h, which is not ideal in critical care settings. Although our group has previously investigated *Aspergillus* using this approach [12], further simplification of the protocol, such as reducing drug concentrations and incubation time, could improve its practical applicability. Most studies to date have focused on azoles and echinocandins [13,19, 20], and expanding this strategy to other antifungal classes, including polyenes, would be an important next step. Overall, studies including larger and more diverse isolate collections will be important to better define the potential of AFST-MS in routine clinical practice.

## Conclusion

Our findings demonstrate that the simplified AFST-MS protocol enables rapid assessment of FLZ susceptibility in *Candida* isolates, showing good overall agreement with the reference method. This approach may serve as a complementary tool to support therapeutic decision-making, particularly in clinical settings where rapid results are critical and for selected *Candida* species. However, performance varied across species, and further validation with larger and more diverse isolate collections is required.

## Supplementary material

10.1099/jmm.0.002172 Supplementary Material 1.

## Abbreviations

AFST: Antifungal susceptibility testing
AFST-MS: MALDI-TOF MS-based antifungal susceptibility testing
CA: categorical agreement
CCI: composite correlation index
CLSI: Clinical and Laboratory Standards Institute
EA: Essential agreement
EUCAST: European Committee on Antimicrobial Susceptibility Testing
FLZ: fluconazole
HCCA: α-cyano-4-hydroxycinnamic acid
I: Intermediary
MALDI-TOF MS: matrix-assisted laser desorption/ionization time-of-flight mass spectrometry
ME: Major error
mE: minor error
MIC: minimum inhibitory concentration
MPCC: minimal profile change concentration
R: Resistant
ROC: receiver operating characteristic
S: Susceptible
SDA: Sabouraud dextrose agar
VME: very major error
