# Supplementary material for: A simplified MALDI-TOF MS method for rapid fluconazole susceptibility testing in Candida species
Source: J Med Microbiol. 2026 Jun 15;75(6):002172. doi: 10.1099/jmm.0.002172 (PMC13268205; doi:10.1099/jmm.0.002172)
Supplement: Supplementary Material 1. [file jmm-75-02172-s001.pdf]

Table S1. Mean values of maximum composite correlation index (CCI) (4–32 µg/mL FLZ) and null CCI (0–4 µg/mL FLZ) obtained by AFST-MS for each *Candida* isolate. Results are expressed as mean ± standard deviation, with *p* values calculated using the unpaired t-test.

		MS-AFST		
**Species**	**Strain designation**	**Maximum CCI (mean±SD)**	**Null CCI (mean±SD)**	***p***
C. albicans (14)	LIF 14297	0.89±0.05	0.70±0.06	0.01
	LIF 14306	0.78±0.04	0.70±0.04	0.04
	LIF 14526	0.94±0.05	0.70±0.06	0.01
	LIF 15234	0.99±0.04	0.74±0.06	0.01
	LIF 12560	0.88±0.0009	0.90±0.006	0.03
	LIF 14112	0.96±0.01	0.81±0.04	0.02
	LIF 14200	0.97±0.04	0.71±0.06	0.04
	LIF 14211	0.92±0.03	0.85±0.02	0.03
	LIF 14004	0.87±0.02	0.72±0.02	0.004
	LIF 15188	0.93±0.03	0.80±0.05	0.11
	LIF 15272	0.97±0.04	0.81±0.05	0.07
	LIF 15355	0.97±0.02	0.82±0.009	0.01
	LIF 16543	0.79±0.04	0.93±0.03	0.04
	LIF E10	0.70±0.006	0.89±0.10	0.17
C. parapsilosis (4)	LIF 14451	0.90±0.01	0.76±0.01	0.001
	LIF 14447	0.92±0.02	0.86±0.02	0.01
	LIF 15134	0.97±0.02	0.80±0.02	0.004
	LIF 16747	0.91±0.02	0.72±0.04	0.01
C. tropicalis (8)	LIF 14464	0.93±0.08	0.70±0.04	0.04
	LIF 14529	0.91±0.02	0.70±0.04	0.01
	LIF 14846	0.92±0.03	0.73±0.03	0.005
	LIF 15292	0.89±0.03	0.70±0.06	0.03
	LIF 16453	0.76±0.05	0.99±0.03	0.01
	LIF 16496	0.62±0.04	0.92±0.05	0.004
	LIF 16924	0.72±0.03	0.89±0.04	0.01
	LIF 16903	0.72±0.03	0.85±0.002	0.03
C. glabrata (10)	LIF 14103	0.77±0.02	0.98±0.01	0.003
	LIF 14119	0.78±0.08	0.98±0.03	0.07
	LIF 14330	0.62±0.10	0.88±0.04	0.04
	LIF 14500	0.81±0.009	0.71±0.006	0.0005
	LIF 14821	0.81±0.005	0.91±0.03	0.03
	LIF 14840	0.70±0.03	0.92±0.05	0.01
	LIF 15888	0.79±0.01	0.89±0.04	0.04
	LIF 16987	0.79±0.01	0.92±0.01	0.001
	LIF 16995	0.31±0.06	0.60±0.08	0.02
	LIF 16574	0.49±0.04	0.68±0.05	0.04
C. auris (9)	LIF 16597	0.92±0.07	0.71±0.02	0.04
	LIF 16605-1	0.88±0.03	0.80±0.02	0.04
	LIF 16605-2	0.92±0.05	0.71±0.04	0.01
	LIF 16606-1	0.89±0.03	0.72±0.03	0.02
	LIF 16606-2	0.97±0.009	0.90±0.01	0.007
	LIF 16607	0.92±0.02	0.82±0.01	0.01
	LIF 16610	0.99±0.01	0.75±0.05	0.02
	LIF 16615	0.94±0.04	0.75±0.07	0.03

	LIF 16619	0.89±0.04	0.71±0.006	0.02
C. krusei (6)	LIF 16152	0.73±0.03	0.89±0.04	0.01
	LIF 16234	0.72±0.03	0.91±0.05	0.02
	LIF 16602	0.72±0.02	0.89±0.01	0.003
	LIF 15557	0.70±0.04	0.89±0.01	0.009
	LIF 15506	0.76±0.0009	0.95±0.03	0.01
	LIF 16395	0.81±0.01	0.98±0.04	0.03
C. albicans ATCC 90028		0.99±0.005	0.88±0.03	0.04
C. parapsilosis ATCC 22019		0.99±0.04	0.73±0.02	0.009
C. krusei ATCC 6258		0.79±0.07	0.98±0.03	0.04

Table S2. Comparison of representative MALDI-TOF MS–based antifungal susceptibility testing (AFST) approaches, including CCI-based MPCC methods, simplified AFST-MS strategies, and machine learning-based models. The table summarizes key differences in incubation time, accuracy, methodological complexity, and potential applicability in routine clinical laboratories.

Method	Principle	Incubation time	Accuracy*	Methodological complexity	Clinical readiness
CCI-based MPCC approach	Full antifungal concentration range with determination of MPCC using CCI	~15 h	High (good correlation with MIC)	High (multiple concentrations, longer workflow)	Moderate
Simplified AFST-MS	Fixed antifungal concentrations with binary classification based on CCI comparison	3 h	Good (CA: 85.2%)	Low (reduced steps and simplified interpretation)	High
Machine learning–based approach (e.g., Delavy et al.)	Classification of spectral patterns using machine learning algorithms	~3 h	~85%	Very high (requires computational modeling and training datasets)	Low to moderate

*Accuracy values are reported as described in the respective studies and may not be directly comparable due to differences in study design, species distribution, and evaluation criteria.
